# *cis* Versus *trans*-Azobenzene: Precise Determination of NMR Parameters and Analysis of Long-Lived States of ^15^N Spin Pairs

**DOI:** 10.1007/s00723-017-0968-8

**Published:** 2017-12-22

**Authors:** Kirill F. Sheberstov, Hans-Martin Vieth, Herbert Zimmermann, Konstantin L. Ivanov, Alexey S. Kiryutin, Alexandra V. Yurkovskaya

**Affiliations:** 1 International Tomography Center SB RAS, Novosibirsk, 630090 Russia; 2 State Scientific Research Institute of Chemistry and Technology of Organoelement Compounds, Moscow, 105118 Russia; 3 Freie Universität Berlin, 14195 Berlin, Germany; 4 Department of Biomolecular Mechanisms, Max-Planck-Institut für Medizinische Forschung, 69120 Heidelberg, Germany; 5 Novosibirsk State University, Novosibirsk, 630090 Russia

## Abstract

We provide a detailed evaluation of nuclear magnetic resonance (NMR) parameters of the *cis*- and *trans*-isomers of azobenzene (AB). For determining the NMR parameters, such as proton–proton and proton–nitrogen *J*-couplings and chemical shifts, we compared NMR spectra of three different isotopomers of AB: the doubly ^15^N labeled azobenzene, ^15^N,^15^N′-AB, and two partially deuterated AB isotopomers with a single ^15^N atom. For the total lineshape analysis of NMR spectra, we used the recently developed ANATOLIA software package. The determined NMR parameters allowed us to optimize experiments for investigating singlet long-lived spin states (LLSs) of ^15^N spin pairs and to measure LLS lifetimes in *cis*-AB and *trans*-AB. Magnetization-to-singlet-to-magnetization conversion has been performed using the SLIC and APSOC techniques, providing a degree of conversion up to 17 and 24% of the initial magnetization, respectively. Our approach is useful for optimizing the performance of experiments with singlet LLSs; such LLSs can be exploited for preserving spin hyperpolarization, for probing slow molecular dynamics, slow chemical processes and also slow transport processes.

**Electronic supplementary material:**

The online version of this article (10.1007/s00723-017-0968-8) contains supplementary material, which is available to authorized users.

## Introduction

Long-lived spin states (LLSs) is an emerging topic of research in modern nuclear magnetic resonance (NMR) [1–5]. Such states are protected by symmetry and relax much slower than longitudinal magnetization. Consequently, LLSs can be used to probe slow molecular motions and slow chemical exchange and also to preserve non-equilibrium spin order. The simplest example of LLS is given by the singlet state of a spin pair [1, 4, 5]: in-pair dipolar relaxation cannot drive singlet–triplet transitions; consequently, singlet spin order relaxes only because of other mechanisms, which are usually less efficient (or even much less efficient). In practice, the ratio of the singlet-state lifetime, $T_{\text{LLS}}$, and the longitudinal relaxation time, $T_{1}$, can reach values much greater than unity. For instance, for the β-CH_2_ protons in partially deuterated amino acids $T_{\text{LLS}} /T_{1}$ can be as large as 45 [6]. In specially designed molecules, lifetimes of LLSs of ^13^C spins in liquids can be as long as 1 h [7].

For exploiting LLSs in NMR spectroscopy and imaging it is necessary to populate them, i.e., to generate singlet spin order from spin magnetization, and to maintain the resulting LLS. For maintenance of the singlet state, one needs to provide experimental conditions such that this state is an eigen-state of the time-independent Hamiltonian of the spin pair. Such conditions are fulfilled [1, 4, 5] when the spin pair becomes strongly coupled so that the difference in NMR frequency, $\delta \nu$, of the two spins becomes smaller than their scalar spin–spin coupling, $J$. In turn, such conditions are fulfilled when the system is brought to a sufficiently low external magnetic field [3] or when a strong spin-locking RF field is applied [2]. A somewhat special case [7–9] is given by molecules where the paired spin is only slightly non-equivalent: in this situation, the condition $J \gg \delta \nu$ is fulfilled even at the high magnetic field of an NMR spectrometer. Using such molecular systems is advantageous because experiments can be run directly inside an NMR spectrometer or MRI scanner; there is also no need to use spin-locking, which can cause unwanted sample heating by RF excitation.

To populate LLSs in strongly coupled spin pairs, special NMR techniques are needed for performing magnetization-to-singlet (M2S) conversion, while the reverse S2M conversion is required for LLS observation. Suitable methods [4] for manipulating singlet spin order in such cases are given by multi-pulse M2S/S2M techniques developed by the Levitt group [10], by spin-locking induced crossing (SLIC) [11–13] or by adiabatic passage spin order conversion (APSOC) proposed by some of us [14–17]. To use these methods, precise knowledge of the NMR parameters of the molecule under study is required for correct setting of the inter-pulse delays in the M2S/S2M method, for optimizing the RF-field strength in the SLIC method and the RF frequency in the APSOC method. When the spin system is not restricted to a spin pair, precise determination of the NMR parameters may become challenging.

**Fig. 1:**
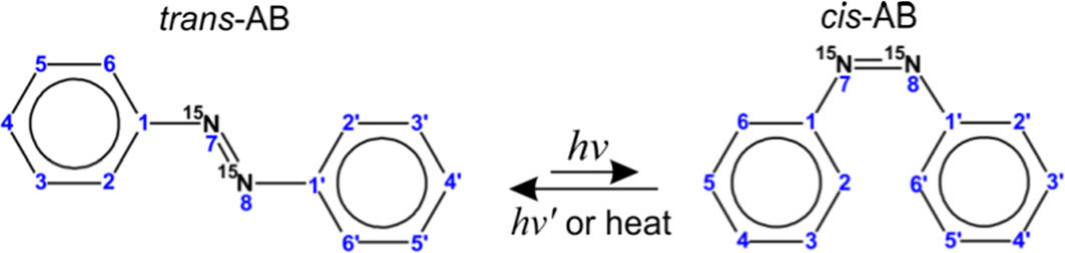
Structures and atom numbering in the studied isotopomer of AB. *trans*-to-*cis* conversion is due to light excitation, $h\nu$; the back *cis*-to-*trans* conversion is thermally activated or it results from light excitation $h\nu '$

As far as systems, in which strongly coupled spin pairs exist even at high fields, are concerned, suitable molecules are the ones containing chemically equivalent but magnetically non-equivalent spins [7–9, 12, 13, 18–20]. In this situation, weak magnetic non-equivalence is necessary for the M2S/S2M conversion (in magnetically equivalent spin pairs such a conversion is not feasible); at the same time, singlet maintenance is possible even at high fields in the absence of spin-locking. In this work, we propose to exploit pairs of ^15^N-nuclei in isotopically labeled azobenzene (^15^N-AB). ^15^N,^15^N′-AB is a symmetric molecule, which can exist in the *cis* and *trans*- forms, see Fig. 1. To be more precise, only the *trans*-isomer, *trans*-AB, is symmetric whereas, strictly speaking, the *cis*-isomer, *cis*-AB, has no geometrical symmetries, even though it seems from Fig. 1 that there are. In reality, the *cis*-AB molecule is not planar and hence not symmetric [21, 22]; otherwise, the protons of the two phenyl rings would overlap with each other. Such overlaps lead to deformation of the molecule also being the reason, why the *cis*-AB is less stable than the *trans*-AB. At room temperature, AB exists in the *trans*-form; however, light excitation, $h\nu$, at a wavelength between 320 and 380 nm, leads to formation of *cis*-AB, which is relatively stable. The back *cis*-to-*trans* conversion takes several days, but it can be made faster by light excitation, $h\nu^{{\prime }}$ with wavelength between 400 and 450 nm [23]. This photo-switching ability of AB has found a wide range of applications to conformational control of various biological systems [24]

The two N-atoms are chemically equivalent in both forms of AB. In both molecules, magnetic non-equivalence is provided by the small difference in nitrogen–proton coupling: each nitrogen atom has different couplings to the protons of the nearby phenyl ring as compared to the protons of the remote phenyl ring. Hence, due to the small imbalance, $\delta J$, in the N–H couplings the two N-atoms form a spin system of the AA′-type instead of A_2_ rendering M2S/S2M conversion feasible. Here we studied protonated ^15^N,^15^N′-AB; in addition, we used data of partially deuterated AB with a single ^15^N atom (see Supplementary Materials). In these compounds, one of the phenyl rings is fully deuterated and the ^15^N atom is adjacent either to the protonated or deuterated phenyl ring.

In this work, we perform precise analysis of NMR spectra of both forms of ^15^N,^15^N′-AB and determine their NMR parameters (scalar spin–spin couplings and chemical shifts). Data from single-labeled partially deuterated AB support this analysis and render data determination more reliable. The knowledge of the NMR parameters allows us to apply M2S/S2M techniques and optimize their performance. Hence, we can demonstrate the presence of singlet LLSs of the ^15^N, ^15^N′ spin pairs and measure their lifetimes in both isomers of AB. Comparison of the $T_{\text{LLS}}$ value in the *cis* and *trans*-forms allows us to reveal the influence of molecular symmetry on the LLS lifetime.

## Methods

### Sample Preparation

NMR spectra were recorded for the following samples: (1) 0.15 M of ^15^N,^15^N′-AB in CD_3_CN; the sample volume was 600 μl; (2) for measuring the singlet-state lifetime, $T_{\text{LLS}}$, 0.5 M of ^15^N,^15^N′-AB in CD_3_CN (i.e., almost saturated solution) in a smaller volume of 300 μl was used to minimize effects of convection [25]. All samples were degassed using several freeze–pump–thaw cycles and sealed by flame.

Synthesis of ^15^N,^15^N′-AB was performed in the following two-stage way. First, ^15^N-nitrobenzene was synthesized. To do so, we used the following procedure. 5.16 g of sodium nitrate-^15^N was added to 75 ml CF_3_COOH followed by 5 g of benzene. The mixture was stirred for 4 h at room temperature. With H_2_O, the mixture was quenched and treated with sodium hydroxide pellets to set pH 10. Saturation with NaCl was followed by extraction with ether performed three times; the combined ether extracts were dried with anhydrous MgSO_4_, and ^15^N-nitrobenzene was distilled in 90% yield.

After that, we synthesized ^15^N,^15^N′-AB using ^15^N-nitrobenzene. To a solution of 6.5 g NaOH in 15 ml water and 50 ml CH_3_OH, we added 5 g of ^15^N-nitrobenzene. To the stirred mixture 6 g zinc-powder (90%) was added and refluxed for 10 h. The mixture was filtered while hot and the precipitate was washed with a small amount of CH_3_OH. By adding concentrated HCl, the pH value was adjusted to 7; then the solution was filtered again and methanol was distilled on the rotary evaporator. Crude labeled AB was purified by chromatography (Silica, mobile phase 8:2 *n*-hexane/CH_2_Cl_2_) and re-crystallized from EtOH. We achieved 80% yield of ^15^N-labeled AB.

### Analysis of NMR Spectra

Temperature stabilization is of high importance for ^15^N spectra, whereas ^1^H signals of azobenzene are not sensitive to slight temperature variation. Temperature stabilization was achieved by flushing a strong flow of dry air on the sample (ca. 500 l/h keeping it by 1 °C warmer than outside). After each experiment we checked that the ^15^N signals stayed at the same resonance frequency—the best indicator of temperature stability. This was fulfilled in all experiments presented here. NMR spectra were recorded at room temperature at magnetic fields $B_{0}$ = 9.4 and 16.4 T (corresponding to 400 and 700 MHz ^1^H NMR frequency, respectively). They were analyzed by total lineshape analysis using the ANATOLIA software package [26]. For local minima suppression, a broadening approach was used [27]. To determine NMR parameters in a precise way, we compared the NMR spectra of the two molecules as shown in Fig. 1 with the NMR data reported earlier by one of us for AB with a single ^15^N label, see Supplementary Materials. Such a comparison helps to determine couplings of the ^15^N-atoms to protons of the close and the remote phenyl rings. Knowing these parameters, we are able to improve the reliability of the NMR analysis of ^15^N,^15^N′-AB.

### SLIC and APSOC

**Scheme 1:**
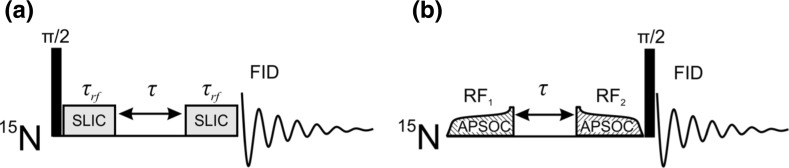
Pulse sequences of the SLIC (**a**) and APSOC (**b**) methods. RF fields are applied to the ^15^N spins of AB. See text for further explanation

Experimental protocols for SLIC and APSOC are shown in Scheme 1.

- In the SLIC method [11], see Scheme 1a, the initial longitudinal magnetization is converted into transverse magnetization by applying a $\pi /2$-pulse on resonance with the ^15^N spins. After that, magnetization is “locked” in the rotating frame using a “SLIC pulse”, i.e., a continuous-wave resonant RF field (the phase of this RF field is shifted by 90°). When the RF-field strength, $\nu_{1}$, matches the *J*-coupling, $J_{\text{NN}}$, of the two ^15^N spins, there is a level anti-crossing in the rotating frame, hence magnetization can be converted into long-lived singlet spin order. At the level anti-crossing coherent spin mixing of the $|S\rangle$ and $|T_{ + } \rangle$ states occurs; thus, M2S conversion is taking place. In practice, coupling with all protons must be taken into account. Therefore, the duration of the RF-excitation period, $\tau_{\text{rf}}$, is optimized to provide the highest M2S/S2M conversion degree. After that the RF field is turned off and the LLS is maintained during the time $\tau$ (as discussed below, singlet maintenance is feasible even without spin-locking). For converting the singlet order back into observable transverse magnetization another RF pulse is applied with $\nu_{1} = J_{\text{NN}}$ and duration equal to $\tau_{\text{rf}}$. After the second SLIC pulse, the free induction decay (FID) signal is taken and Fourier transformed to obtain the NMR spectrum. For probing the LLS lifetime, the delay $\tau$ is stepwise varied.
- In the APSOC method [15], see Scheme 1b, the M2S conversion is performed using the RF_1_ field with time-dependent amplitude, $\nu_{1} (t)$, monotonously increasing from zero to $\nu_{1}^{ \hbox{max} } > J_{\text{NN}}$; the frequency, $\nu_{\text{rf}}$, of this switched RF field needs to be carefully chosen to provide the desired spin order conversion (the offset, $\delta \nu_{\text{rf}}$, of the RF frequency from the “center of the spectrum” has to be non-zero [15]). Again, for singlet order maintenance during the variable time period $\tau$ spin-locking is not used. Finally, S2M conversion is performed by the RF_2_ field, which is reduced to zero in an adiabatic fashion, and the resulting magnetization is recorded using a $\frac{\pi }{2}$ NMR pulse to obtain the FID signal and the NMR spectrum. In the APSOC method, longitudinal magnetization is converted into singlet order and back, whereas the SLIC method is dealing with transverse magnetization. In this work, both methods are used for generating and observing LLSs. Further details of the method can be found in Refs. [15, 16]. To get rid of background signals, we also combined APSOC with the method, termed singlet order selection (SOS) filter [16], for suppressing background signals from longitudinal magnetization. We also used a phase cycle for SLIC as described before [16], which enables SOS filtering for SLIC. In the filtering procedure, we used phases of the SLIC pulses and of the receiver as given by Table 1.

**Table 1:** Phase settings used for SOS filtering by SLIC

First SLIC pulse	Second SLIC pulse	Receiver
$x$	$x$	$x$
$x$	$-\, x$	$-\, x$
$- \,x$	$-\, x$	$x$
$-\, x$	$x$	$- \, x$

In both methods, the RF pulses are applied to the ^15^N spins. SLIC and APSOC experiments were done for both *trans*-AB and *cis*-AB. As already mentioned above in the present case, LLS maintenance is possible without applying an RF field because of the tiny magnetic non-equivalence of the two ^15^N-atoms in AB, i.e., $\delta \nu \ll J_{\text{NN}}$. As a consequence, the singlet state of the spin pair of the two ^15^N-spins is nearly an eigen-state of the system, i.e., it is maintained even in the absence of RF fields, which are, in most other cases, necessary for maintaining LLSs.

The optimum conditions for adiabatic passage were determined from numerical simulations for a two-spin system with the *J*-coupling equal to $J_{\text{NN}}$ and the difference in Larmor frequency, $\delta \nu ,$ taken equal to the difference in scalar coupling, $\delta \nu = \delta J = (J_{\text{H2N7}} - J_{\text{H2N8}} )$, since $\delta J$ describes the magnetic inequivalence of the two ^15^N-spins in the AB molecule. The coupling constants *J*_H6N7_ have the same value as *J*_H2N7_ for both *cis*- and *trans*-AB because of the molecular symmetry; moreover, because of the averaging over the fast rotation of the phenyl rings around the C1–N7 bond *J*_H6N8_ is equal to *J*_H2N8_. The same is true for the meta-protons. For taking into account effects of all other protons, in both techniques, SLIC and APSOC, the S2M/M2S efficiency was additionally optimized by adjusting the RF-field strength and experimental timings.

## Results and Discussion

In this section, we present a detailed NMR analysis of AB necessary for optimization of the relevant parameters for SLIC and APSOC. Finally, we apply the results of this optimization to probe LLS in AB. SLIC and APSOC experiments presented in this section were done at two $B_{0}$ fields, 9.4 and 16.4 T.

### Analysis of NMR Spectra

**Fig. 2:**
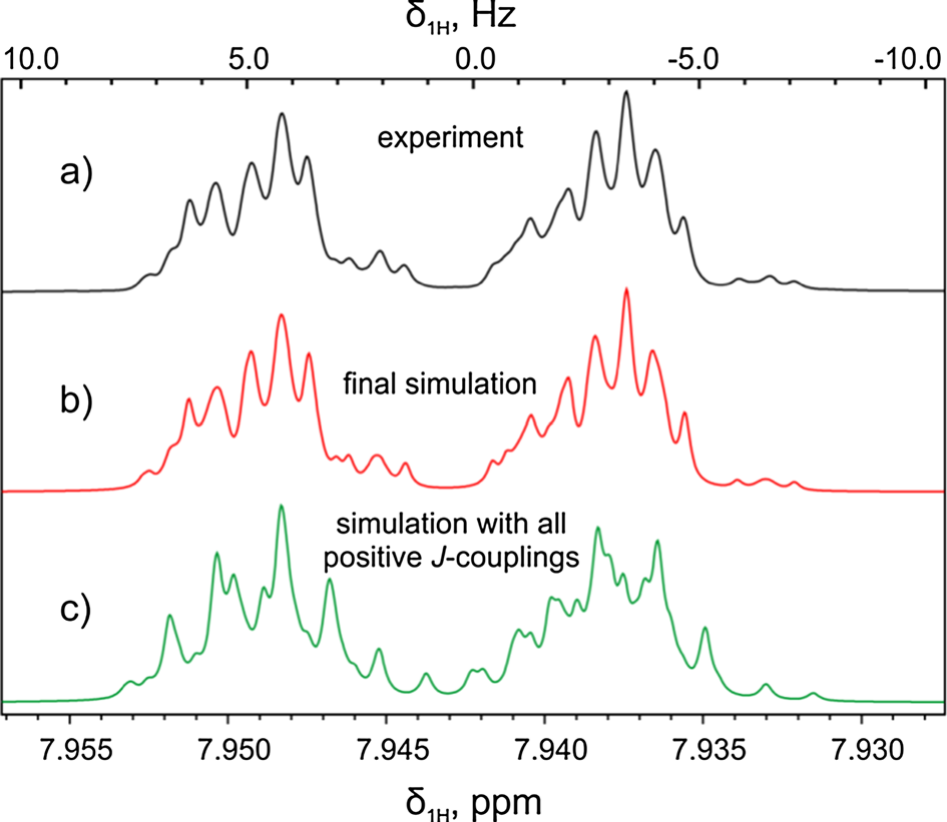
700 MHz ^1^H NMR spectrum of the ortho-proton multiplets of *trans*-AB (sample 1) (*a*), simulation of the spectrum (*b*), simulation of the spectrum obtained after optimization, but taking all $J_{\text{NH}}$-couplings positive

**Fig. 3:**
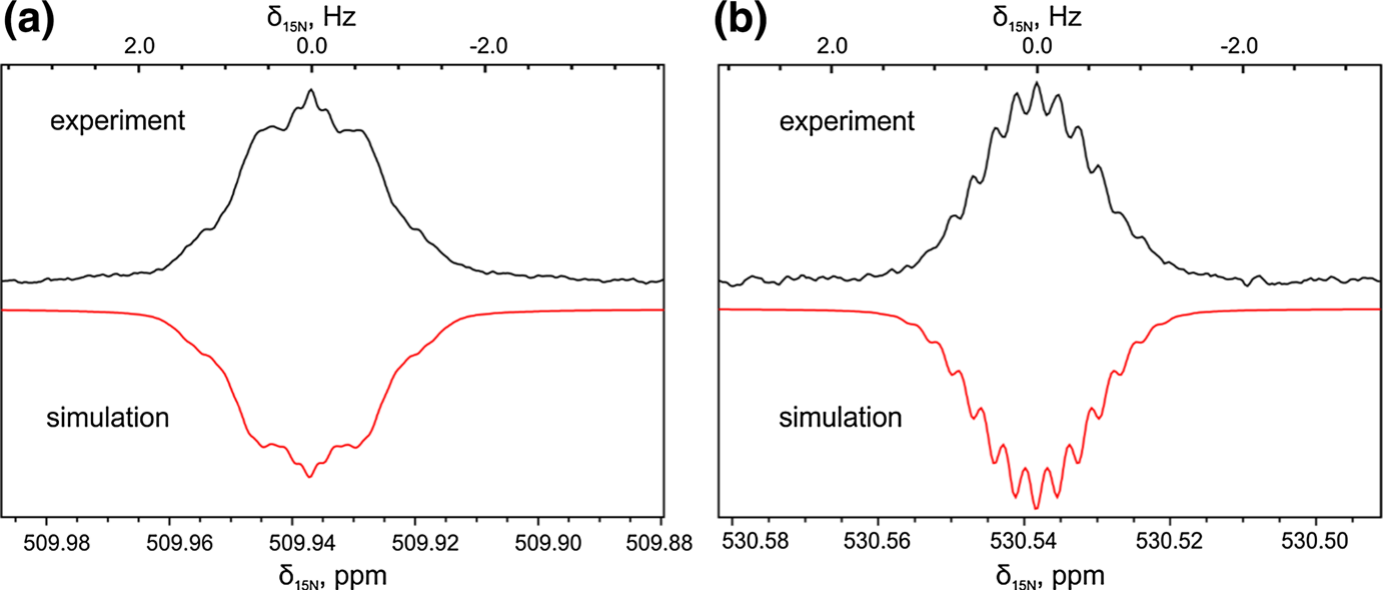
^15^N NMR spectra of *trans*-AB (sample 1) (**a**) and *cis*-AB (**b**), taken at 16.4 T; simulation of the spectra is added for comparison. For convenience of the reader, the phase of the simulated spectra is inverted

Proton spectra of AB in its *trans*-form are shown in Fig. 2. We are able to achieve excellent agreement between experiments and simulations. Importantly, in our simulations we varied both the size and the sign of all spin–spin interactions. From Fig. 2, one can clearly see that the shape of NMR multiplets depends on the sign of proton–nitrogen couplings, $J_{\text{NH}}$: variation of sign of the couplings [26] allowed us to achieve perfect agreement with the experimental spectra. Analysis of the proton spectra was accompanied by simulations of the nitrogen spectra, see Fig. 3: the spectra were fitted with a common set of parameters providing very good agreement for the ^1^H and ^15^N spectra of AB. From the analysis, we are able to determine the nitrogen–nitrogen coupling, $J_{\text{NN}}$, which is equal to 16.2 and 21.8 Hz for the *trans*-AB and *cis*-AB, respectively.

The analysis was performed for both forms of AB. As one can see from Fig. 3, the ^15^N spectra of *trans*-AB and *cis*-AB have a different appearance; consequently, NMR parameters are significantly different: not only is the $J_{\text{NN}}$ coupling different, but also the $J_{\text{NH}}$ couplings.

The results of the total line-shape analysis are summarized in Table 2.

**Table 2:** *J*-coupling constants of AB given in Hz as determined from ^1^H and ^15^N spectral analysis of AB

Nuclei	H2	H3	H4	H5	H6	N7	N8	H2′	H3′	H4′	H5′	H6′
H2		7.96 (7.96)	1.22 (1.17)	0.58 (0.56)	2.11 (2.19)	1.67 (1.05)	− 0.42 (− 0.27)	0.0 (0.0)	0.0 (0.0)	0.0 (0.0)	0.0 (0.0)	0.0 (0.0)
H3			7.37 (7.47)	1.53 (1.41)	0.58 (0.56)	0.20 (0.27)	0.16 (0.18)	0.0 (0.0)	0.0 (0.0)	0.0 (0.0)	0.0 (0.0)	0.0 (0.0)
H4				7.37 (7.47)	1.22 (1.17)	0.31 (0.0)	− 0.32 (− 0.38)	0.0 (0.0)	0.0 (0.0)	0.0 (0.0)	0.0 (0.0)	0.0 (0.0)
H5					7.96 (7.96)	0.20 (0.27)	0.16 (0.18)	0.0 (0.0)	0.0 (0.0)	0.0 (0.0)	0.0 (0.0)	0.0 (0.0)
H6						1.67 (1.05)	− 0.42 (− 0.27)	0.0 (0.0)	0.0 (0.0)	0.0 (0.0)	0.0 (0.0)	0.0 (0.0)
N7							16.2 (21.8)	1.67 (1.05)	0.20 (0.27)	0.31 (0.0)	0.20 (0.27)	1.67 (1.05)
N8								− 0.42 (− 0.27)	0.16 (0.18)	− 0.32 (− 0.38)	0.16 (0.18)	− 0.42 (− 0.27)
H2′									7.96 (7.96)	1.22 (1.17)	0.58 (0.56)	2.11 (2.19)
H3′										7.37 (7.47)	1.53 (1.41)	0.58 (0.56)
H4′											7.37 (7.47)	1.22 (1.17)
H5′												7.96 (7.96)
H6′												
*δ* (ppm)	7.925 (6.847)	7.591 (7.301)	7.557 (7.191)	7.591 (7.301)	7.925 (6.847)	509.94 (530.54)	509.94 (530.54)	7.925 (6.847)	7.591 (7.301)	7.557 (7.191)	7.591 (7.301)	7.925 (6.847)

Parameters for *trans*-AB are given in the first lines of the table cells; parameters for *cis*-AB are given in brackets in the second line of the table cells. In the bottom row, also chemical shifts of the nuclei are given in ppm

Finally, we measured the *T*_1_-relaxation of the ^15^N spins in AB and found *T*_1_ = 10.1 and 19.5 s at *B*_0_ = 9.4 T for *trans*-AB and *cis*-AB, respectively. At *B*_0_ = 16.4 T, *T*_1_ = 3.5 and 6.9 s are obtained for *trans*-AB and *cis*-AB, respectively. All relaxation times were measured for sample 2. They are significantly shorter than those found previously [28] (about 60 s for *trans*-AB) at a lower field of 2.1 T; faster relaxation at higher fields can be explained by contributions from chemical shift anisotropy, which increase quadratic with the magnetic field strength.

The NMR parameters determined here provide data for optimization of the experimental settings for SLIC and APSOC. Specifically, the level anti-crossing in the rotating frame is expected when $\nu_{1}$ matches $\left| {J_{\text{NN}} } \right|$, whereas the conversion frequency is given by the effective $\delta J$ values.

### SLIC Optimization

Optimization of the SLIC parameters was done by systematic variation of $\nu_{1}$ and $\tau_{\text{rf}}$. When performing optimization we also introduced a spin-locking RF field on the nitrogen channel between the SLIC pulses: this RF field was applied to the nitrogen spins with the aim to suppress their thermal spin magnetization, which partly recovers due to *T*_1_-relaxation and affects the resulting signal. Such a saturating RF field does not disturb the singlet spin order, i.e., it does not affect the NMR signals we are interested in. The strength of the spin-locking RF field was 100 Hz; it was applied during the whole time $\tau$ between the SLIC pulses. We also used SOS filtering to suppress any background signals. Here, we present optimization only for *trans*-AB; optimization for *cis*-AB can be performed in a similar way.

**Fig. 4:**
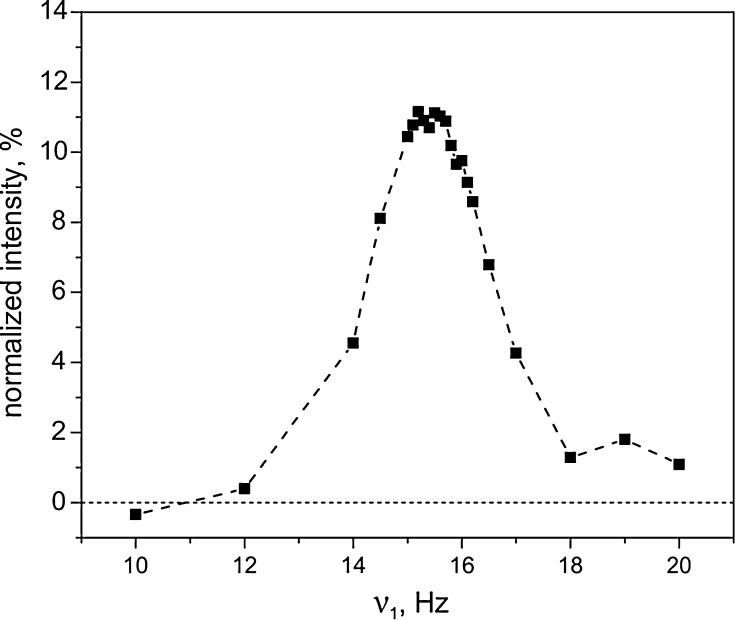
Optimization of the SLIC-pulse amplitude, $\nu_{1}$, for generating the LLS in *trans*-AB. Duration of the SLIC-pulse was $\tau_{rf} = 0.3$ s, the delay between the two SLIC pulses was $\tau = 10$ s. Here the time $\tau$ was fixed, and we assume the same relaxation contribution to each of the measured points, $B_{0} = 16.4$ T. The signal intensity is given in percent of the thermal signal of *trans*-AB

**Fig. 5:**
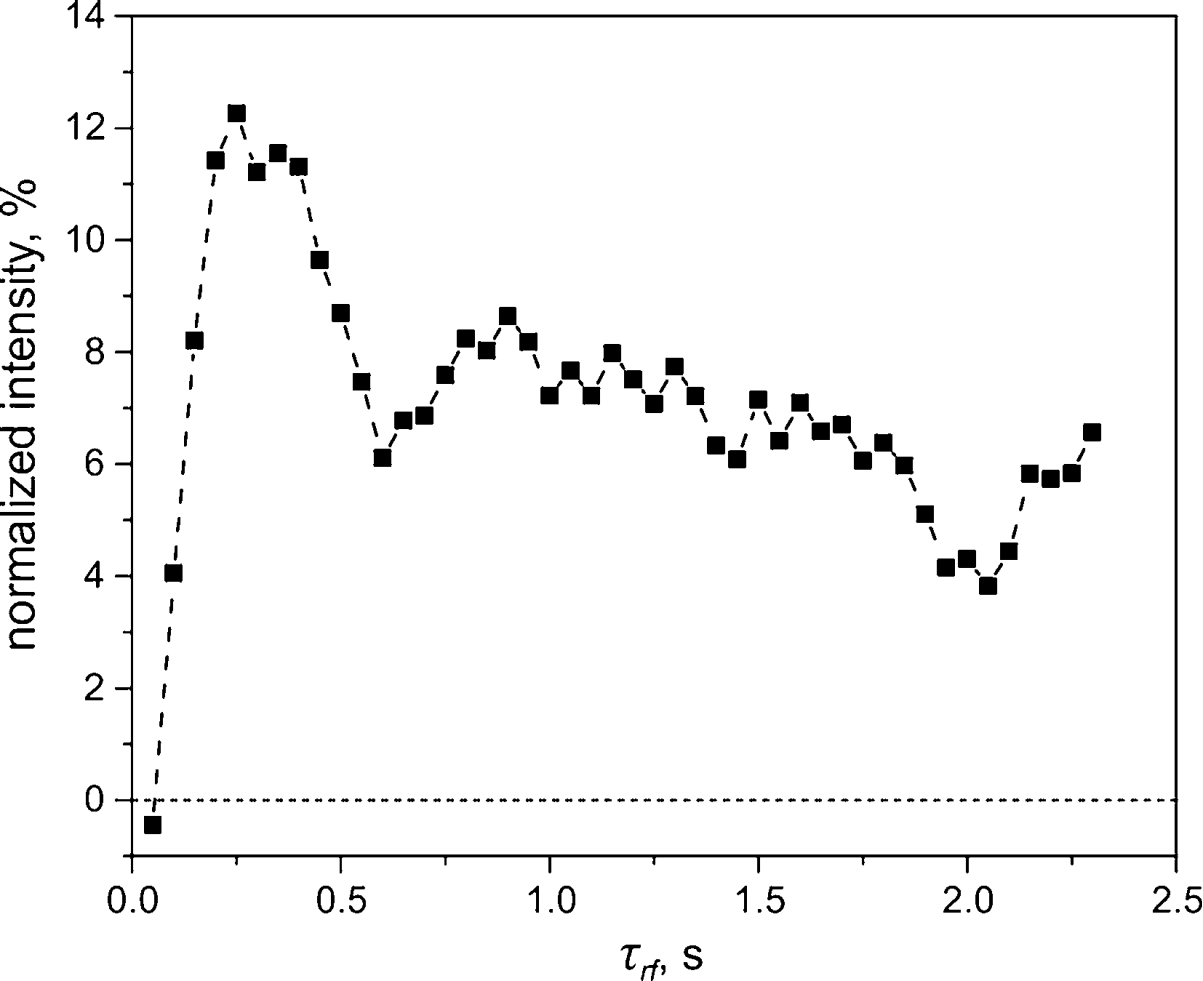
Optimization of the SLIC-pulse duration, $\tau_{\text{rf}}$, for generating the LLS in *trans*-AB. The SLIC-pulse amplitude was $\nu_{1} = 16$ Hz, the delay between the two SLIC pulses was $\tau = 3$ s. We applied a 100 Hz spin locking field during $\tau$, which was enough to saturate the thermal signal during these 3 s, $B_{0} = 16.4$ T. The signal intensity is given in percent of the thermal signal of *trans*-AB

The results of optimization of the SLIC experiment are presented in Figs. 4 and 5. As one can see, the $\nu_{1}$-dependence of the resulting NMR signal has a pronounced peak centered at $\nu_{1} \approx J_{\text{NN}}$. This is in agreement with the simple consideration that SLIC requires matching of the two interactions to fulfill the conditions for level crossing in the rotating frame.

When such a matching is achieved, perturbation terms can induce singlet–triplet mixing, required for generating the LLS. In the case under study, the perturbation is due to the small magnetic non-equivalence of the two ^15^N-spins coming from $\delta J \ne 0$. In SLIC, singlet–triplet conversion occurs in a coherent fashion; hence, the $\tau_{\text{rf}}$-dependence of the signal is expected to contain oscillations. Such oscillations are indeed seen in the $\tau_{\text{rf}}$-dependence, see Fig. 5. With time, the oscillations are damped because the precise $\delta J$ value depends on the state of the proton spins, which is different in different molecules: while in the simplest case of only one proton there should be oscillations with a single frequency equal to $J_{\text{NH}} /\sqrt 2$, in a multi-proton system there is a distribution of frequencies causing destructive interference of the quantum beats. Consequently, the spread of $\delta J$ [caused by different $J_{\text{NH}}$ couplings with the *ortho*- (H2, H6), *meta*- (H3, H5), and *para*- (H4) protons] results in damping of the observed oscillations.

The efficiency of generating an LLS, which we achieved here using SLIC, is 9% at *B*_0_ = 9.4 T and 17% at 16.4 T. The exact method for determining the conversion efficiency is discussed in more detail in Sect. 3.4. The optimal parameters determined for SLIC are: $\tau_{\text{rf}} = 0.3$ s, $\nu_{1} = 16$ Hz (for *trans*-AB) and $\tau_{\text{rf}} = 0.3$ s, $\nu_{1} = 22$ Hz (for *cis*-AB).

### APSOC Optimization

In addition to SLIC, we also performed APSOC experiments and determined the optimal $\nu_{1}^{ \hbox{max} }$ and $\tau_{\text{rf}}$ values. As in the SLIC case, when performing optimization we introduced an RF field to the ^15^N-spins between the RF_1_ and RF_2_ fields. The strength of this RF field was 100 Hz; it was applied during the whole time $\tau$ between the APSOC RF fields. We also used the SOS filter to suppress any background signals. Here we present details of optimization only for *trans*-AB.

**Fig. 6:**
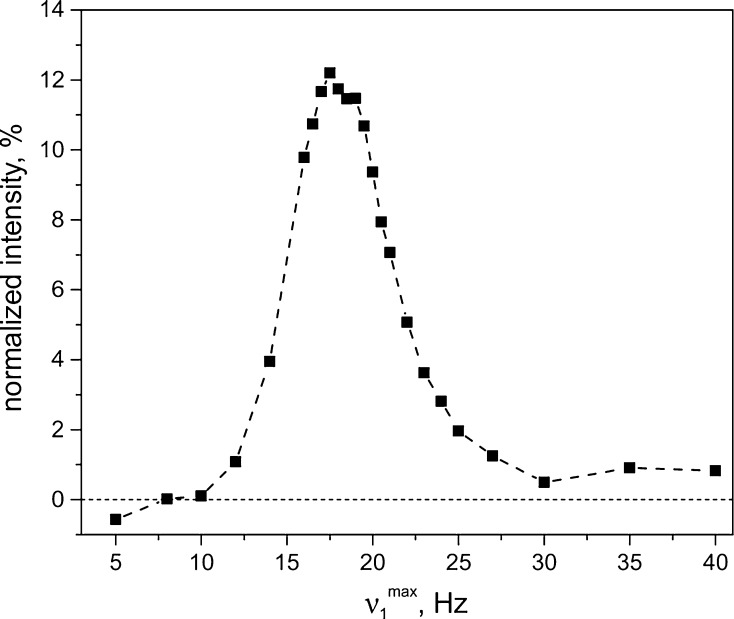
Optimization of $\nu_{1}^{ \hbox{max} }$ in APSOC in *trans*-AB. Here, the offset of the RF frequency from the center of the ^15^N multiplet, $\delta \nu_{\text{rf}}$, was set to $\pm \ 10$ Hz. The delay between the two adiabatic passages was $\tau = 20$ s, RF-switching time $\tau_{\text{rf}} = 1.5$ s, $B_{0} = 9.4$ T. The signal intensity is given in percent of the thermal signal of *trans*-AB

Optimization of $\nu_{1}^{ \hbox{max} }$ is presented in Fig. 6. The dependence peaks at $\nu_{1}^{ \hbox{max} } \approx 18$ Hz, which is slightly above $J_{\text{NN}}$. Hence, as expected for APSOC, it is necessary to go above $J_{\text{NN}}$ to pass through the level crossing. Further increase of $\nu_{1}^{ \hbox{max} }$ results in reduction of the NMR signal because the level crossing is passed at a faster speed incompatible with adiabatic variation of the spin Hamiltonian (in the experiments the total switching time is kept the same for all $\nu_{1}^{ \hbox{max} }$ values).

**Fig. 7:**
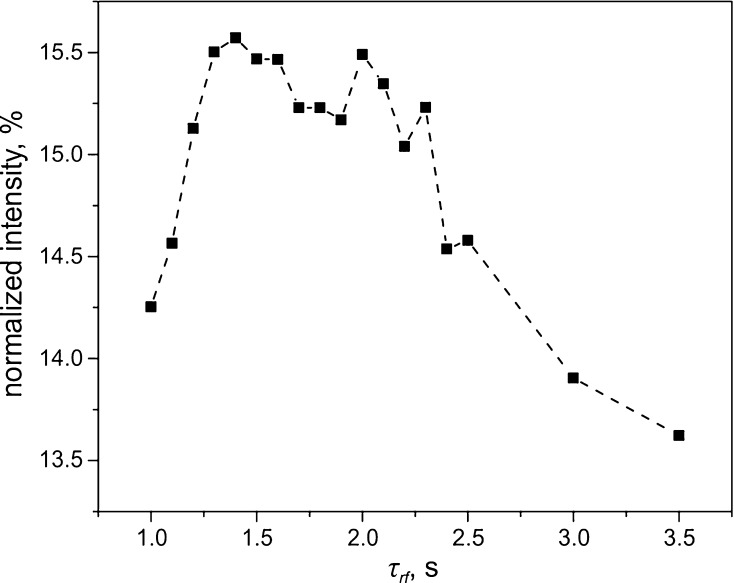
Optimization of the time, $\tau_{\text{rf}}$, of adiabatic passage in APSOC for *trans*-AB. Here $\nu_{1}^{ \hbox{max} }$ was 18 Hz, the offset frequency, $\delta \nu_{\text{rf}}$, from the center of the ^15^N multiplet was set to $\pm \ 9.5$ Hz, $B_{0} = 9.4$ T. The signal intensity is given in percent of the thermal signal of *trans*-AB

In addition, we optimized the time, $\tau_{\text{rf}}$, of the RF ramps, see Fig. 7. The $\tau_{\text{rf}}$-dependence of the resulting NMR signal has a maximum. The reason is that short $\tau_{\text{rf}}$ times are incompatible with adiabatic variation of the spin Hamiltonian, while at long $\tau_{\text{rf}}$ times relaxation during RF switching comes into play and reduces the spin order.

The efficiency of generating an LLS, which we achieved here is 15% for the 9.4 T spectrometer and 24% for 16.4 T using APSOC; see further discussion in Sect. 3.4. The different level of conversion efficiency is related to the inhomogeneity of the RF field, which is by a factor of two smaller for the probe of the 16.4 T NMR spectrometer as compared to that of the 9.4-T spectrometer. Note, that the APSOC method is less sensitive to RF-field inhomogeneity, compared to the SLIC method, as follows from comparison of the conversion efficiency found at 9.4 and 16.4 T. Furthermore, APSOC provides a higher conversion efficiency. The optimal parameters determined for APSOC are: $\tau_{\text{rf}} = 1.5$ s, $\delta \nu_{\text{rf}} = \pm 10$ Hz, $\nu_{1}^{ \hbox{max} } = 18$ Hz (for *trans*-AB), $\tau_{\text{rf}} = 3.0$ s, $\delta \nu_{\text{rf}} = \pm 7$ Hz, $\nu_{1}^{ \hbox{max} } = 25$ Hz (for *cis*-AB). The optimized time-profile of the RF pulse as well as the optimization parameters are shown in Supplementary Materials. The time-profile was calculated for a two-spin system with an effective chemical shift difference of 2.1 Hz (which equals to difference $(J_{\text{H2N7}} - J_{\text{H2N8}} )$ and a J-coupling equal to 16 Hz ($J_{\text{NN}}$). It was calculated to make an RF pulse with a constant adiabaticity for the given system using APSOC GUI. The profile itself is also shown at Scheme 1b.

### LLS Measurements

Having determined the optimal parameters for SLIC and APSOC we can study the LLS of the ^15^N spin pair in both forms of AB by varying the delay $\tau$. We measured the resulting signals as a function of $\tau$ and fitted the signal using the following expression:

$$ I(\tau ) = I_{0} + A_{1} \exp \left[ { - \frac{\tau }{{t_{\text{short}} }}} \right] + A_{2} \exp \left[ { - \frac{\tau }{{t_{\text{long}} }}} \right]. $$

**Fig. 8:**
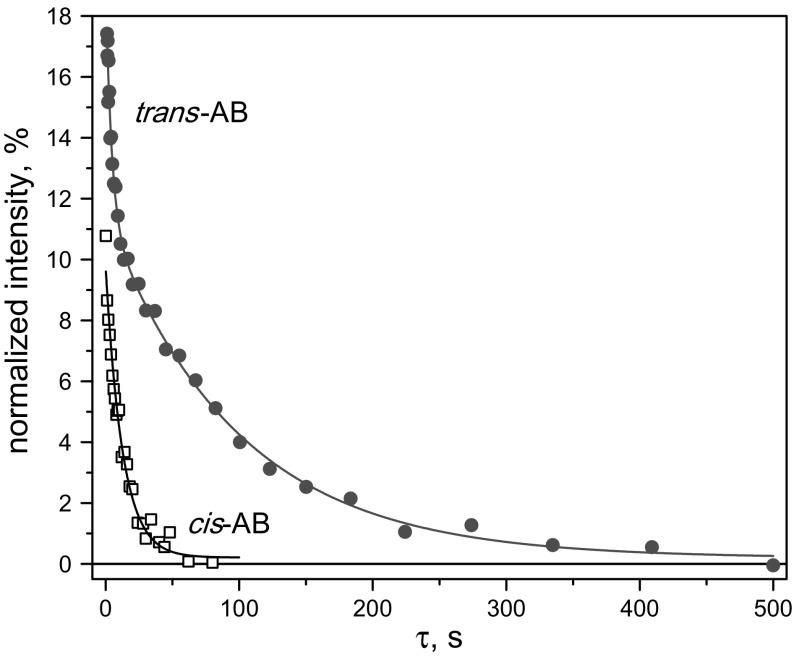
$\tau$-Dependence of the NMR signal of *trans*-AB (red circles) and *cis*-AB (black squares) in the SLIC experiment. The $\tau$-dependences of the resulting signal were fitted by a bi-exponential function, see text, the parameters are: $I_{0} = 0.19\%$, $A_{1} = 7.6\%$, $A_{2} = 11.3\%$, $t_{\text{short}} = 4.3$ s, $t_{\text{long}} = 98$ s (for *trans*-AB); $I_{0} = 0.2\%$, $A_{2} = 9.5\% ,A_{1} = 0\%$, $t_{\text{long}} = 12.7$ s (for *cis*-AB). The experiment was done at $B_{0} = 16.4$ T; SLIC parameters are $\nu_{1} = 16$ Hz, $\tau_{rf} = 0.3$ s. The signal intensity is given in percent of the thermal signal of the corresponding AB

Hence, we used bi-exponential fitting and determined the two relaxation times, $t_{\text{short}}$ and $t_{\text{long}}$, corresponding to the triplet relaxation time and LLS lifetime, respectively. The amplitudes, $A_{1}$ and $A_{2}$, stand for the weights of the components. Since the signal, $I(\tau )$, is measured in units of thermal polarization, the $A_{2}$ value gives the efficiency of generating the LLS. Generally, determination of parameters at bi-exponential fitting is known to be an ill-posed problem; however, in the case $T_{\text{LLS}} = t_{\text{long}} \gg t_{\text{short}}$ (which applies to our experiments) the parameters are determined in a reasonably reliable way. We fitted the black curve in Fig. 8 by a single exponent, fixing one of the amplitudes to zero. Actually, this fitting behavior allows us to draw the important conclusion that the relaxation time of the ^15^N singlet state in *cis*-AB almost coincides with *T*_1_.

LLS measurements using the SLIC method are presented in Fig. 8 for both *trans*-AB and *cis*-AB. One can clearly see that the LLS lifetime is much longer for the *trans*-isomer, as it is expected from symmetry considerations [29]. Specifically, at $B_{0} = 16.4$ T the LLS lifetimes are 98 s and 13 s for *trans*-AB and *cis*-AB, respectively. The achieved conversion efficiencies given by the $A_{2}$ value are 11 and 9.5% for *trans*-AB and *cis*-AB, respectively. Hence, the optimization performed here allows us to generate and study LLS in both forms of AB. Our experiments also show a remarkably strong effect of the molecular structure on the LLS lifetime. At 16.4 T the $T_{\text{LLS}} /T_{1}$ ratio is 28 and 1.9 for *trans*-AB and *cis*-AB, respectively. The same $T_{\text{LLS}}$ was obtained by APSOC for the *trans*-isomer.

**Fig. 9:**
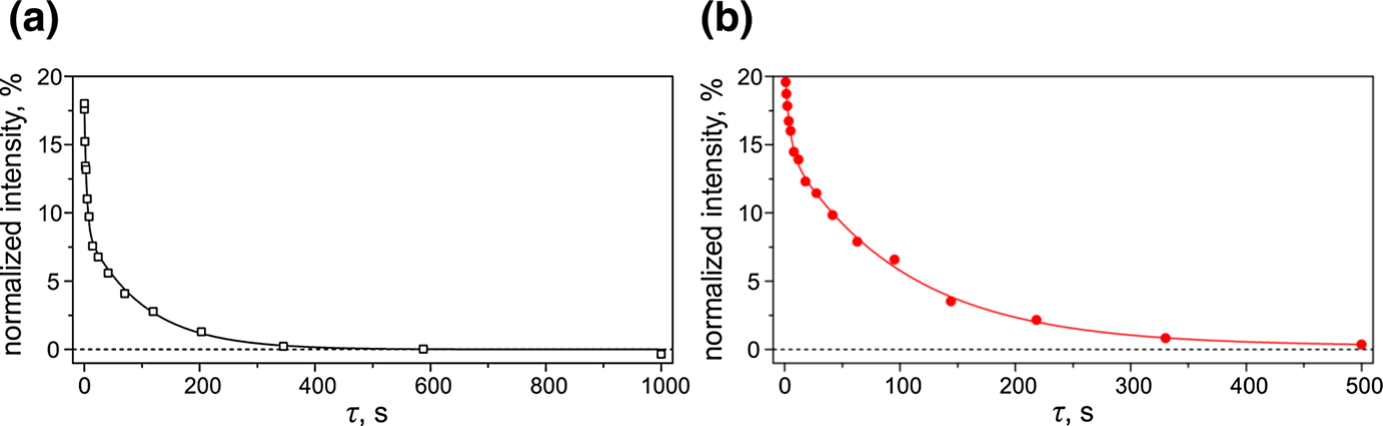
$\tau$-Dependence of the NMR signal of *trans*-AB in the SLIC (**a**) and APSOC (**b**) experiments. The $\tau$-dependences of the resulting signal were fitted by a bi-exponential function, see text, the parameters are: $I_{0} = 0$, $A_{1} = 9.0\%$, $A_{2} = 8.8\%$, $t_{\text{short}} = 3.9$ s, $t_{\text{long}} = 101$ s (for SLIC); $I_{0} = 0$, $A_{1} = 6.1\%$, $A_{2} = 14.5\%$, $t_{\text{short}} = 4.1$ s, $t_{\text{long}} = 103$ s (for APSOC). The experiment was done at $B_{0} = 9.4$ T; SLIC-parameters are $\nu_{1} = 16$ Hz, $\tau_{\text{rf}} = 0.3$ s; APSOC parameters are: $\tau_{\text{rf}} = 1.5$ s, $\delta \nu_{\text{rf}} = \pm 10$ Hz, $\nu_{1}^{ \hbox{max} } = 18$ Hz. The signal intensity is given in percent of the thermal signal of *trans*-AB

In the context of this work, it is of interest to compare the performance of the two methods, SLIC and APSOC, used for generating LLSs. Such a comparison is presented in Fig. 9. Both experiments provide access to the same LLS, as follows from identical relaxation times $t_{\text{long}}$ determined in both experiments. At the same time, APSOC has a better performance as far as the M2S/S2M conversion efficiency. Indeed, the $A_{2}$ value is a factor of 1.6 higher in the APSOC experiment. The reason is that in the SLIC case the conversion is more sensitive to the experimental timing. Because of the coherent character of the conversion, see Fig. 5, different timing is optimal for different $\delta J$ values. Consequently, the optimized $\tau_{\text{rf}}$ value does not provide maximal conversion efficiency for molecules in different states of protons. APSOC is much less affected by this problem because adiabatic passage through level crossing provides the same conversion efficiency for all $\delta J$ values once the RF ramp is beyond a certain threshold value (which is compatible with adiabatic RF switching). We expect that in simple systems of two nearly equivalent spins (when $\delta \nu$ has the same value for all molecules) both methods would have exactly the same performance. However, when there is inhomogeneity of both the $B_{0}$ and RF fields over the sample using APSOC is preferable, as it is less sensitive to small variations of $\nu_{1}$ and $\delta \nu$. For this reason, in the present case, we also gain from using the APSOC method.

## Conclusions

In summary, we presented a detailed study and precise determination of NMR parameters of AB in its *trans*- and *cis*-form. We were able to determine precisely the $J_{\text{NN}}$ coupling as well as $J_{\text{NH}}$ couplings to the protons of the close-by and remote phenyl rings. Such an analysis has provided the experimental parameters required for generating LLSs in these molecules by means of the APSOC and SLIC techniques. By applying these methods, we probed LLSs and measured their lifetimes. The efficiency of the M2S/S2M conversion is ca. 1.5 times larger for APSOC as compared to SLIC (for both *cis*-AB and *trans*-AB, at *B*_0_ = 9.4 and 16.4 T); the better performance of APSOC is explained. We obtained strongly increased lifetimes of the singlet spin order in *trans*-AB; a $T_{\text{LLS}} /T_{1}$ ratio of 28 is achieved, whereas for *cis*-AB this ratio drops to 1.7.

We anticipate that our results are useful for improving the efficiency of generating and detecting singlet-state LLSs, in particular, in systems containing “nearly equivalent” spins. Such LLSs can be exploited in NMR applications where RF power exerted on the sample is an important concern because singlet maintenance is feasible even without applying RF fields. In addition, using the techniques outlined here, one can probe the impact of molecular structure on LLS lifetimes.

## Electronic supplementary material

Below is the link to the electronic supplementary material.

Supplementary material 1 (DOCX 344 kb)
