# Supplementary material for: cis Versus trans-Azobenzene: Precise Determination of NMR Parameters and Analysis of Long-Lived States of 15N Spin Pairs
Source: Appl Magn Reson. 2017 Dec 22;49(3):293–307. doi: 10.1007/s00723-017-0968-8 (PMC5811614; doi:10.1007/s00723-017-0968-8)
Supplement: Supplementary file 1 — Supplementary material 1 (DOCX 344 kb) [file 723_2017_968_MOESM1_ESM.docx]

**Supplementary Materials for the article**

***Cis* versus *trans*-Azobenzene: Precise determination of NMR parameters and analysis of long-lived states of ^15^N spin pairs**

Kirill F. Sheberstov,^1,2^ Hans-Martin Vieth,^1,3^ Herbert Zimmermann,^4^ Konstantin L. Ivanov,^1,5^

Alexey S. Kiryutin,^1,5^ Alexandra V. Yurkovskaya^1,5,^*

*^1^ International Tomography Center SB RAS, Novosibirsk, 630090, Russia*

*^2^ State Scientific Research Institute of Chemistry and Technology of Organoelement Compounds, Moscow, 105118, Russia*

*^3^ Freie Universität Berlin, Berlin, 14195, Germany*

*^4^ Max-Planck-Institut für Medizinische Forschung, Heidelberg, 69120, Germany*

*^5^ Novosibirsk State University, Novosibirsk, 630090, Russia*

* Corresponding author; e-mail: [yurk@tomo.nsc.ru](mailto:yurk@tomo.nsc.ru)

This file contains additional NMR data of several isotopomers of *trans* and *cis* azobenzenes and the shape of RF pulse used for APSOC.

Here we present NMR spectra of partially deuterated azobenzene (AB) with a single ^15^N atom (compounds II and III, see **Chart 1S**). In compounds II and III one of the phenyl rings is deuterated; in compound II the ^15^N atom is adjacent to the protonated phenyl ring, while in compound III it is adjacent to the deuterated phenyl ring.


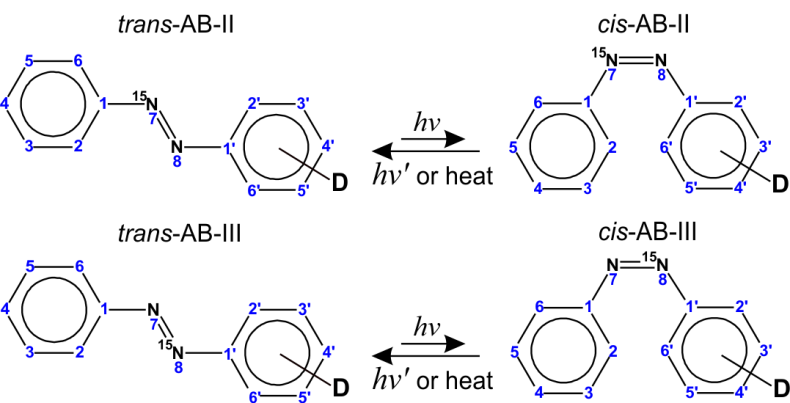


**Chart 1S.** Structures and atom numbering in the studied isotopomers of AB. *Trans-*to*-cis* conversion is due to light excitation, $h\nu$; the back *cis-*to*-trans* conversion is thermally activated or it is due to light excitation $h\nu'$.

**Sample preparation.** Compounds II and III were synthesized by V. A. Chertkov et al. [1],[2]. Here we present NMR spectra of the following samples: 600 µl of 0.1 M of compound II in CD_3_CN; 600 µl of 0.1 M of compound III in CD_3_CN.


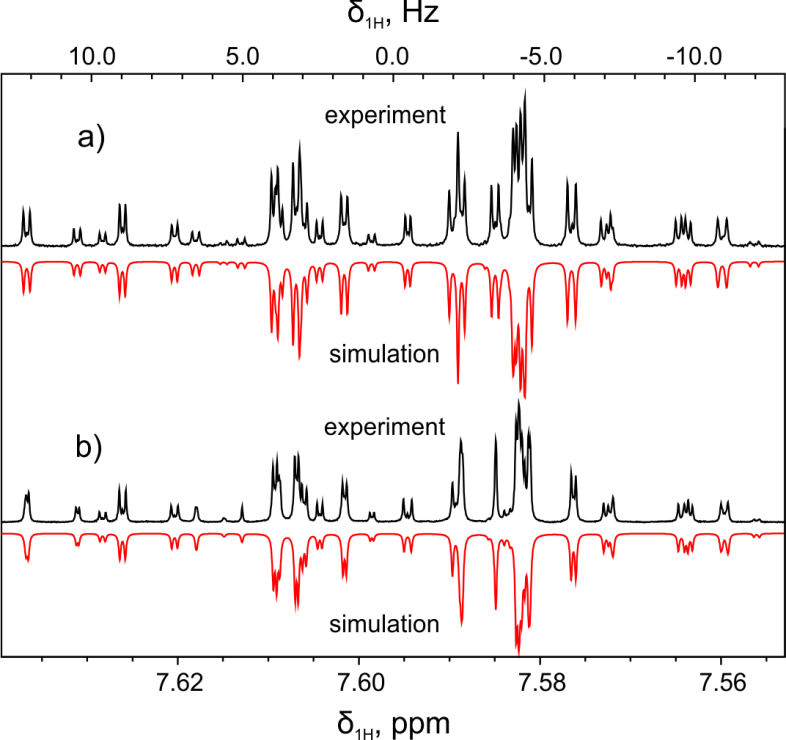


**Figure 1S**. 300 MHz ^1^H NMR spectra of *trans*-AB-II (a) and *trans*-AB-III (b). Here we show the signals of the meta- and para- protons of the phenyl rings. For convenience of the reader, the phase of the simulated spectra is inverted.

**NMR spectra.** NMR spectra were taken using a 300 MHz NMR spectrometer and simulated using the same method as described in the main text of the article. Spectra were recorded and analyzed for both *cis*-AB and *trans*-AB. Such an analysis allowed us to specify $J_{NH}$ couplings and to discriminate the couplings of the ^15^N spins to the adjacent and remote phenyl rings.

Typical proton NMR spectra are shown in **Figure 1S** for *trans*-Azobenzene (for both compound II and compound III). One can see that the simulation method indeed allows one to achieve perfect agreement between the measured and calculated spectra. Specifically, positions of NMR lines as well as structure of NMR multiplets are perfectly reproduced. Together with NMR data for doubly ^15^N-labeled AB (shown in the main text of the article) these results enable determination of all NMR parameters, shown in **Table 2**.

Figure S2. The shape of the optimized RF pulse for APSOC as described in [3], [4].

**References**:

[1] Pushkareva, A. A.; Shestakova, A.K.; Chertkov, V. A. “15N NMR spectral parameters for structure elucidation and conformational analysis: Indole, quinoline and azobenzene derivatives” Abstracts of Papers of 243rd ACS National Meeting & Exposition, San Diego, CA, United States, March 25-29, 2012, Pages ORGN-413 Conference; Meeting Abstract; Computer Optical Disk 2012  CODEN: 69PCV3

[2] V. A. Chertkov, D. A. Cheshkov, T. A. Ganina et al. “Novel high resolution NMR techniques for elucidation of molecular solution-state structure and dynamics”, Abstracts of the International Symposium and Summer School in Saint Petersburg “Nuclear Magnetic Resonance in Condensed Matter”, 11th Meeting “NMR in Life Sciences”. Saint Petersburg University Press (Saint Petersburg University), 2014 – P. 11.

[3] Kiryutin, A. S.; Pravdivtsev, A. N.; Yurkovskaya, A. V.; Vieth, H.-M.; Ivanov, K. L. Nuclear Spin Singlet Order Selection by Adiabatically Ramped RF Fields. J. Phys. Chem. B 2016, 120, 11978-11986.

[4] Pravdivtsev, A. N.; Kiryutin, A. S.; Yurkovskaya, A. V.; Vieth, H.-M.; Ivanov, K. L. Robust conversion of singlet spin order in coupled spin-1/2 pairs by adiabatically ramped RF-fields. J. Magn. Reson. 2016, 273, 56-64.
